# Prostaglandin-E2 Mediated Increase in Calcium and Phosphate Excretion in a Mouse Model of Distal Nephron Salt Wasting

**DOI:** 10.1371/journal.pone.0159804

**Published:** 2016-07-21

**Authors:** Manoocher Soleimani, Sharon Barone, Jie Xu, Saeed Alshahrani, Marybeth Brooks, Francis X. McCormack, Roger D. Smith, Kamyar Zahedi

**Affiliations:** 1 Center on Genetics of Transport, University of Cincinnati College of Medicine, Cincinnati, OH, United States of America; 2 Departments of Internal Medicine, University of Cincinnati College of Medicine, Cincinnati, OH, United States of America; 3 Department of Pharmacology and Cell Biophysics and, University of Cincinnati College of Medicine, Cincinnati, OH, United States of America; 4 Department of Pathology and Laboratory Medicine, University of Cincinnati College of Medicine, Cincinnati, OH, United States of America; 5 Research Services, Veterans Affairs Medical Center, Cincinnati, OH, United States of America; University of Pittsburgh School of Medicine, UNITED STATES

## Abstract

Contribution of salt wasting and volume depletion to the pathogenesis of hypercalciuria and hyperphosphaturia is poorly understood. Pendrin/NCC double KO (pendrin/NCC-dKO) mice display severe salt wasting under basal conditions and develop profound volume depletion, prerenal renal failure, and metabolic alkalosis and are growth retarded. Microscopic examination of the kidneys of pendrin/NCC-dKO mice revealed the presence of calcium phosphate deposits in the medullary collecting ducts, along with increased urinary calcium and phosphate excretion. Confirmatory studies revealed decreases in the expression levels of sodium phosphate transporter-2 isoforms a and c, increases in the expression of cytochrome p450 family 4a isotypes 12 a and b, as well as prostaglandin E synthase 1, and cyclooxygenases 1 and 2. Pendrin/NCC-dKO animals also had a significant increase in urinary prostaglandin E2 (PGE-2) and renal content of 20-hydroxyeicosatetraenoic acid (20-HETE) levels. Pendrin/NCC-dKO animals exhibit reduced expression levels of the sodium/potassium/2chloride co-transporter 2 (NKCC2) in their medullary thick ascending limb. Further assessment of the renal expression of NKCC2 isoforms by quantitative real time PCR (qRT-PCR) reveled that compared to WT mice, the expression of NKCC2 isotype F was significantly reduced in pendrin/NCC-dKO mice. Provision of a high salt diet to rectify volume depletion or inhibition of PGE-2 synthesis by indomethacin, but not inhibition of 20-HETE generation by HET0016, significantly improved hypercalciuria and salt wasting in pendrin/NCC dKO mice. Both high salt diet and indomethacin treatment also corrected the alterations in NKCC2 isotype expression in pendrin/NCC-dKO mice. We propose that severe salt wasting and volume depletion, irrespective of the primary originating nephron segment, can secondarily impair the reabsorption of salt and calcium in the thick ascending limb of Henle and/or proximal tubule, and reabsorption of sodium and phosphate in the proximal tubule via processes that are mediated by PGE-2.

## Introduction

The role of salt wasting and volume depletion in the pathogenesis of hypercalciuria and hyperphosphateuria is poorly understood. Age associated derangements in renal ion transport machinery is well documented, and is important in salt wasting and volume depletion in the elderly [1, 2]. A monogenic disorder associated with primary salt wasting, volume depletion, and hypercalciuria is Bartter’s Syndrome [3]. One of the most severe forms of Bartter’s Syndrome is caused by mutations in the sodium/potassium/chloride co-transporter 2 (NKCC2) in the thick ascending limb of Henle (TALH) [3, 4], and is associated with impairment of calcium reabsorption due to the loss of a favorable electrical gradient [3–5]. Neonatal Bartter’s Syndrome is used synonymously with Hyperprostaglandin E syndrome (HPS) and is characterized by enhanced renal and systemic generation of prostaglandin E2 (PGE-2), which is thought to be largely responsible for the aggravation of its associated clinical symptoms [6–8]. Hypercalciuria, impaired urinary concentrating ability in the face of volume depletion, metabolic alkalosis and the activation of the renin angiotensin system are the most salient features of neonatal Bartter’s Syndrome/HPS [6, 8].

In Pendrin/NCC double KO (pendrin/NCC-dKO or dKO) mice, impaired salt wasting in the distal convoluted tubule (DCT) and the cortical collecting duct (CCD) causes severe volume depletion that is disproportionate to the predicted magnitude of salt wasting that results from impairment of salt reabsorption in these nephron segments [9]. Based on these studies, we hypothesize that salt wasting, irrespective of the primary originating nephron segment, and subsequent activation of prostaglandin synthesis can impair tubular function in various nephron segments, including the proximal tubule (PT), TALH and the collecting duct (CD). Studies presented here establish a link between the initial salt wasting/vascular volume depletion with the impaired electrolyte and water reabsorption in multiple nephron segment. They further demonstrate that salt wasting followed by volume depletion can reduce calcium and phosphate reabsorption and precipitation of calcium phosphate crystals in the CD.

## Materials and Methods

### Animal models

Slc26a4 KO (pendrin-KO), the thiazide sensitive Na/Cl co-transporter KO (NCC-KO) and pendrin/NCC-dKO mice have been previously described [9–11]. Animals used in these studies were cared for following the guidelines and in accordance with a protocol approved by the Institutional Animal Care and Use Committee (IACUC) at the University of Cincinnati. Animals had access to food and water *ad libitum*, were housed in humidity, temperature, and light/dark controlled rooms, and were inspected daily. During the course of these studies animals were not subjected to procedures that cause pain and discomfort. For harvesting of blood and tissue samples, animals were sacrificed using an over dose of sodium pentobarbital (0.8 mg) administered through intraperitoneal (i.p.) injection. These studies were designed and conducted based on ARRIVE Guidelines [12, 13].

High salt diet studies were performed for a period of 7 days during which animals were either placed on liquid 0.25% NaCl (control) diet or the same diet supplemented with 7% NaCl (high salt). The basal liquid diet, TD.06315, was obtained from Envigo-Teklad, Madison, WI. The composition of TD.06315 is as follows: Casein 200, L-cystine 3, maltodextrin 529.486, sucrose 100, soybean oil 70, cellulose 39, Mineral Mix-AIN-93G-MX 35, Vitamin Mix-AIN-93-VX 10, choline bitartarate 2.5, TBHQ-antioxidant 0.014 and xanthan gum 11g/kg). Indomethacin (Sigma-Life Science) was administered by daily i.p. injection (25mg/kg/day) for 3 days. HET0016 (20mg/kg/day) was given through daily i.p. injection for 3 days.

For balance studies, age matched male WT and knockout mice on C57BL/C background (n = 6/group) were housed in metabolic cages and had free access to rodent chow and water. Food intake, water intake, and urine volume were measured daily. For the harvest of tissues, animals were euthanized with the use of excess pentobarbital sodium, according to institutional guidelines and approved protocols.

Tail DNA genotyping for NCC KO, pendrin KO and pendrin/NCC double KO mice. The primers used for genotyping of NCC KO mice were: KO forward, AGG GTC AAG GGC ACG GTT GGC; KO reverse, GGT AAA GGG AGC GGG TCC GAG G; KO rev pk, GCA TGC TCC AGA CTG CCT TG. The PCR conditions were as follows: initial denature for 2 min at 94°C, followed by 35 cycles of 94°C, 30s; 68°C 1 min.

The primers for genotyping of pendrin-KO mice were the same as used in our previous publications reporting the generation of pendrin-KO mice [10]: pair one: PDS A2, GGC AGG CAA GCA TTC TAC CAC TAA G; PDS F7, GGA ACT TCG CTA GAC TAG TAC GCG TG; pair two: PDS-A2-1, GCA GGC AAG CAT TCT ACC AC; PDS-3-S, AGG TAA GAT GCT GCT GGA TAG G. The PCR conditions were as follows: initial denature for 2 min at 94°C, followed by 35 cycles of 94°C, 30sec; 65°C, 30 sec; 68°C 2 min.

### Immunofluorescence microscopy studies

Immunofluorescence microscopic analyses were performed as previously described [9, 14, 15] using antibodies against prostaglandin E synthase1 (Ptges1, sc-20771, 1:50 dilution; Santa Cruz Biotechnology Inc.), Cyclooxygenases 1 & 2 (Cox-1, sc-1754, 1:100 dilution; Cox-2, sc-1747, 1:100 dilution; Santa Cruz Biotechnology Inc.), and sodium phosphate transporter-2 (NaPi-II) isotypes a, b (generous gifts from Dr. H. Murer) and c (sc-163164, 1:50 dilution; Santa Cruz Biotechnology Inc.). Two different antibodies against NKCC2 (monoclonal antibody AT33901a, 1:40 dilution, Abgent, and a polyclonal antibody generated in our laboratory against amino acids 109–129 of the rat NKCC2, 1:200 dilution) were used in our studies with identical results.

### Preparation of kidney extracts and western blot analysis

Flash frozen kidneys were pulverized, washed with ice-cold PBS and subjected to centrigugation at 7,000g for 5 min. Supernatants were discarded and 200 μl of extraction buffer (45 mM HEPES, 0.4 M KCl, 1 mM EDTA, 0.1 mM dithiothreitol, 10% glycerol, pH 7.8) was added to each pellet. Resulting suspensions were mixed vigorously, snap frozen in liquid nitrogen and immediately thawed. Next, 30 μl of 1% Triton X-100 in extraction buffer was added to a 100 μl aliquot of each sample. Samples were mixed vigorously and incubated for 5 min on ice. After centrifugation at 14,000g for 5 min at 4°C to remove cellular debris, the supernatants were collected. The protein contents of kidney and cell extracts were determined by BCA assay (Thermo Scientific, Rockford, IL). For analysis of protein expression levels, 30μg of each extract was size fractionated by polyacrylamide gel electrophoresis, transferred to nitrocellulose membrane and subjected to western blot analysis as previously described using antibodies against prostaglandin E synthase1 (Ptges1, sc-32589, 1:1000 dilution; Santa Cruz Biotechnology Inc.), Cyclooxygenases 1 & 2 (Cox-1, sc-1754, 1:1000 dilution; Cox-2, sc-1747, 1:1000 dilution; Santa Cruz Biotechnology Inc.) [16].

### Analysis of blood chemistry and urine electrolyte composition

Urine electrolyte levels were measured using a urine electrolyte analyzer (EasyLyte Urine Analyzer). Calcium and phosphate levels were measured using commercial kits (BioChain Institute, Inc.). Concentrations of blood Na^+^, K^+^, Ca^++^, and HCO_3_^-^ were measured using i-STAT^R^-1 analyzer with i-STAT *EG7+* cartridges (Abbott Laboratories).

### Biological Assays

Measurement of parathyroid hormone (PTH), fibroblast growth factor-23 (FGF-23) and active vitamin D were performed using USCN Life Science Inc. PTH and FGF-23 assays, and SunRed Biotechnology Vitamin D assay respectively.

### RNA isolation and northern blot analysis

Total cellular RNA was extracted from mouse kidney cortex and medulla as well as small intestine according to established methods [9], quantitated spectrophotometrically, and stored at -80°C. Total RNA from each sample (20 μg/lane) was size fractionated on a 1.2% agarose-formaldehyde gel, transferred to Magna NT nylon membranes, cross-linked by UV light, and baked. PCR generated cDNA fragments specific for NaPi-IIa, NaPi-IIb, tumor necrosis factor-α (TNF-α) and cytochrome p450 4a isotypes 12a (Cyp4a12a) were labeled with ^32^P and used for Northern blot analyses. Hybridization was performed according to established methods. The membranes were washed, blotted dry, and exposed to a PhosphorImager screen (Molecular Dynamics, Sunnyvale, CA). The signal strength of hybridization bands was quantitated by densitometry using ImageQuaNT software (Molecular Dynamics, Sunnyvale, CA).

### Quantitative real time PCR (qRT-PCR) for analysis of NKCC2 isotype expression profile

Total RNA isolated from kidneys of WT and pendrin/NCC-dKO mice was used for first strand DNA synthesis. qRT-PCR for quantitation of NKCC2 isoforms and glycerol phosphate dehydrogenase (GAPDH) was performed using the SYBR^®^ Green Real-Time PCR Master Mix (Life Technologies). The following oligonucleotide primers that were previously utilized for qRT-PCR analysis of NKCC2 isotype specific transcripts were used: NKCC2-A antisense primer, 5’-CCC AGT GAT AGA GGT TAC CAT GGT-3’; NKCC2-B antisense primer, 5’-GAC AAA CCT GTG ATG GCT GTC A-3’; NKCC2-F antisense primer, 5’-ACA ACT ACG CTC AGG CCA ATG-3’; NKCC2 sense primer, 5’-GCC TCT CCT GGA TTG TAG GAG AA-3’ [17]. NKCC2 isoform mRNA expression results were normalized against glyceraldehyde 3-phosphate dehydrogenase (GAPDH) mRNA expression of the corresponding samples. The final results were expressed as fold change in the expression of each NKCC2 isoform in pendrin/NCC-dKO and various treatment groups compared to WT mice.

### PGE-2 measurements

Urinary PGE-2 levels were determined using the Prostaglandin E Metabolite EIA kit (Cayman Chemical Co.).

### 20-HETE measurements

The content of 20-HETE in renal extracts was determined using the 20-HETE ELISA, kit following the manufacturers protocol (Detroit R & D, Inc.).

### Statistical analysis

Results are presented as means ± SEM. Statistical significance (P<0.05) between samples was determined by Student's unpaired *t*-test or ANOVA.

## Results

### Pendrin/NCC-dKO mice develop calcium and phosphate wasting and renal calcium deposits

**Fig 1:**
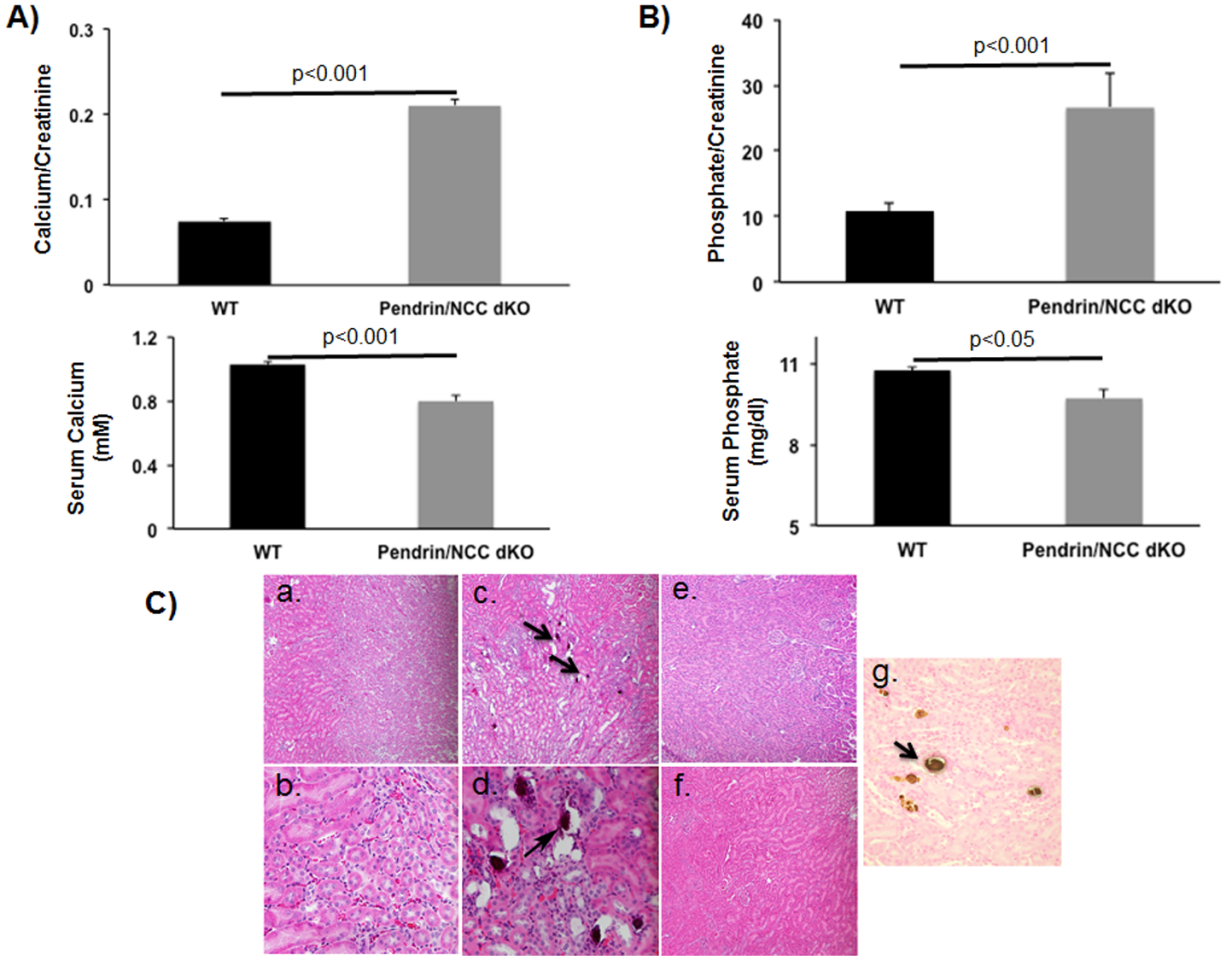
Examination of the effect of simultaneous ablation of pendrin and NCC on renal function and histology of double knockout (pendrin/NCC-dKO) mice. The urinary (top panels) and serum (bottom panels) calcium **(A)** and phosphate **(B)** levels of WT and pendrin/NCC-dKO animals were compared. The results are average +/- SEM of 3 animals/genotype, similar results were obtained in two independent studies. **(C)** Histological examination of the H&E stained kidney sections of WT (panels **a** and **b**, 200X and 400X magnification respectively) and pendrin/NCC-dKO (panels **c** & **d**, 200X and 400X magnification respectively; arrows point to calcium deposits), NCC KO (panel **e**, 200X magnification) and pendrin KO (panel **f**, 200X magnification) mice. Von Kossa staining of kidneys of pendrin/NCC-dKO mice indicates that the deposits contain calcium (panel **g**, 400X magnification, arrow points to Von Kossa stain positive calcium deposit).

The urine calcium measurements (Fig 1A) revealed a 3-fold increase in calcium excretion in dKO compared to WT-animals (0.07+/-0.012 vs. 0.21+/-0.023 calcium/creatinine, p<0.001). Based on previous studies it is known that NCC-KO mice have reduced calcium excretion; whereas, pendrin-KO mice are only mildly hypercalciuric [11, 18]. Phosphate excretion also increased by 2.5 fold (Fig 1B) in dKO mice compared to their WT counterparts (26.6+/-2 vs 10.8+/-0.5 phosphate/creatinine, p<0.001). Serum calcium and phosphate levels were reduced by approximately 22% and 10%, respectively, in pendrin/NCC-dKO mice compared to WT mice (Fig 1A & 1B).

Histological examination (Fig 1C) of the Hematoxylin & Eosin (H&E) stained kidney sections showed multiple calcium deposits in the lumen of medullary collecting ducts and juxtaglomerular apparatus (JGA) hypertrophy in pendrin/NCC-dKO (panels **c & d**, arrows point to calcium deposits) but not in WT mice (panels **a & b**). NCC-KO or pendrin-KO mice do not exhibit any calcium deposits in their kidneys [11, 18] (panels **e & f**). The stones were not visible under polarized light and strongly stained with Von Kossa reagent, indicating that they are composed of calcium and phosphate (panel **g**, arrow points to stained calcium deposit).

### Molecular basis of hypercalciuria and hyperphosphaturia in pendrin/NCC-dKO mice

Next, we examined the serum content of PTH, FGF-23 and active vitamin D (1,25-dihydroxy vitamin D or calcitrol), since these molecules play important roles in the regulation of calcium and phosphate levels [19–24]. Our results indicate that the serum levels of PTH (WT; n = 6: 32.62+/-10.0 pg/ml vs. pendrin/NCC-dKO; n = 6: 20.7+/-6.7 pg/ml), FGF 23 (WT; n = 6: 212.9+/-29.5 pg/ml vs. pendrin/NCC-dKO; n = 6: 229.9+/-42.4 pg/ml) and active vitamin D (WT; n = 6: 114.2+/-2.6 pg/ml vs. pendrin/NCC-dKO; n = 6: 124.7+/-7.3 pg/ml) were not significantly different in WT and pendrin/NCC-dKO mice. The results presented thus far indicate that pendrin/NCC-dKO mice exhibit severe calcium and phosphate wasting, and that the aforementioned changes are independent of the activity of PTH and FGF-23.

Our published results [9] indicate that the extent of salt wasting in pendrin/NCC-dKO mice is disproportionate to the predicted magnitude of impairment in salt reabsorption caused by the deficiency of pendrin and NCC. Based on the above results and the presence of severe calcium and phosphate wasting in pendrin/NCC-dKO mice we examined the potential changes in sodium, chloride, phosphate and calcium transporters in other nephron segments (e.g. proximal tubule and TALH).

**Fig 2:**
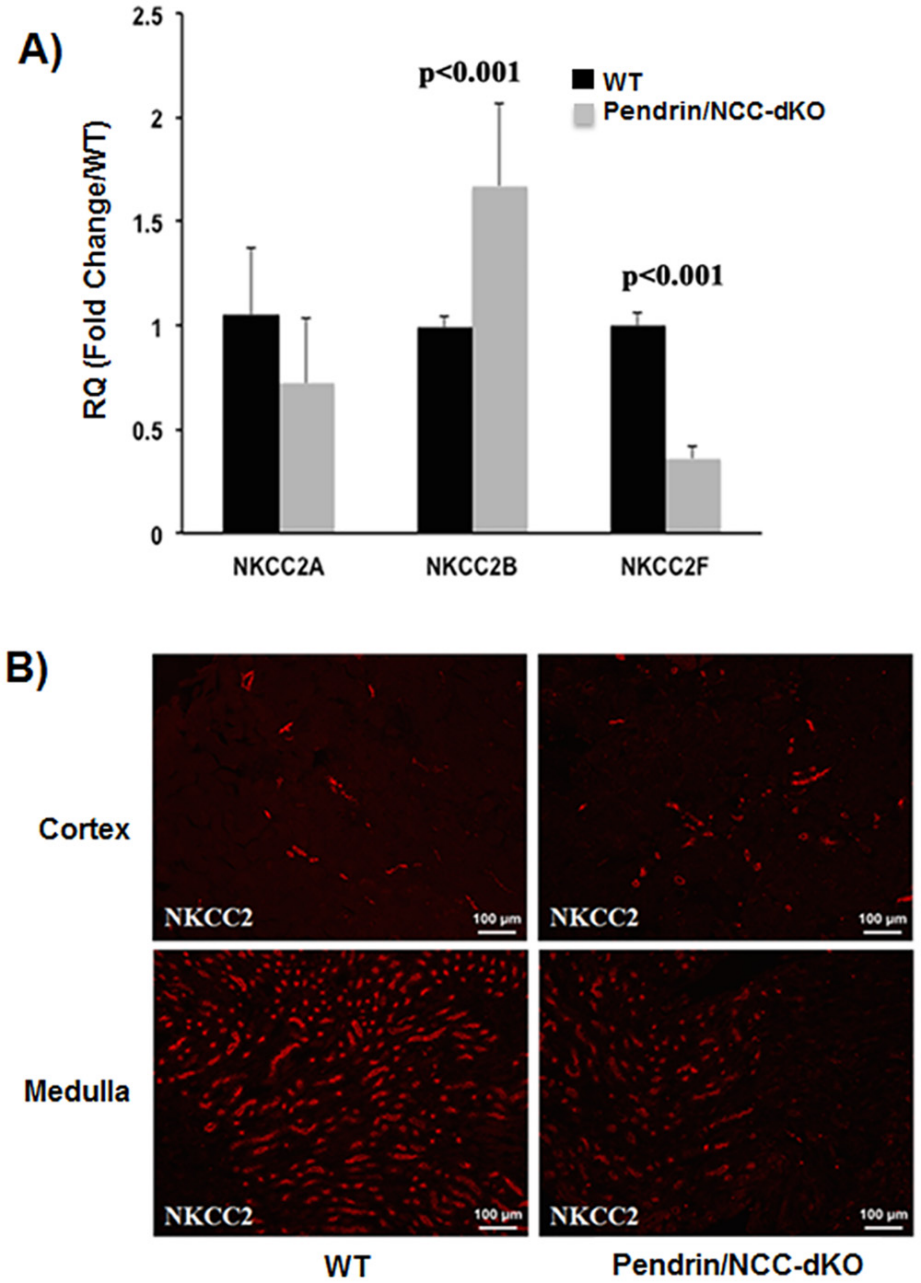
Comparison of the expression of Na-K-2Cl co-transporter 2 (NKCC2) isoforms in the kidneys of WT and pendrin/NCC-dKO mice. **A)** The NKCC2 isoform profiles of WT and pendrin/NCC-dKO mice were compared using qRT-PCR (the results represent the average +/- SEM of n = 4 animals/genotype). **B)** Immunofluorescence microscopic examination of NKCC2 expression in the renal cortex and medulla of WT and pendrin/NCC-dKO mice.

First, we examined the expression of NKCC2, the main salt absorbing transporter in the TALH. In addition to salt reabsorption, NKCC2 plays a significant role in calcium reabsorption via generation of electrical gradient that is favorable to paracellular transport of calcium ions from the lumen of TALH [25]. Examination of the renal expression of NKCC2 isoforms by qRT-PCR indicate that compared to WT mice, the expression of NKCC2F, which is primarily expressed in medullary TALH and plays a significant role in paracellular reabsorption of calcium [25] is significantly decreased; while, the expression of NKCC2-B (expressed in the cortical TALH, [5, 26]) is increased in kidneys of pendrin/NCC-dKO mice (Fig 2A). The expression of NKCC2-A was not significantly different in the WT and pendrin/NCC-dKO animals (Fig 2A). Immunofluorescence microscopic analysis of WT and pendrin/NCC dKO mouse kidneys with NKCC2 antibody revealed that while the intensity of NKCC2 staining in the cortical region of WT and pendrin/NCC-dKO mice are similar, the intensity of NKCC2 staining in the medullary region of the pendrin/NCC-dKO mice is significantly attenuated (Fig 2B). Since A and F isoforms are the predominant medullary subtypes of NKCC2, these results lend support to the qRT-PCR data, which indicate that compared to WT mice the expression of NKCC2F is significantly decreased in medullary TALH in pendrin/NCC-dKO animals.

**Fig 3:**
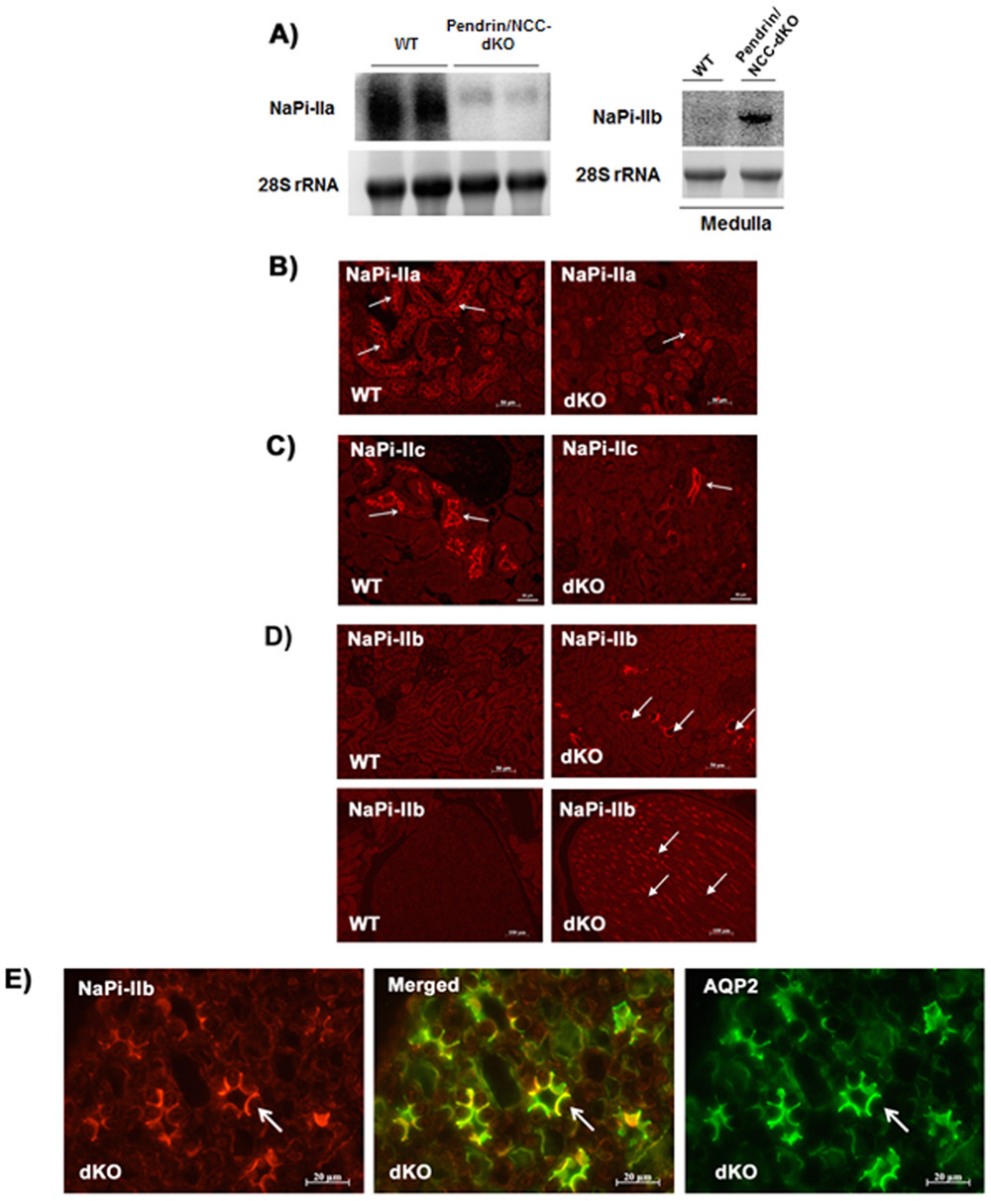
Comparison of the expression of NaPi-II isoforms in the kidneys of WT and pendrin/NCC-dKO mice. **(A)** Northern blot images of NaPi-IIa in kidneys of WT and pendrin/NCC dKO mice (left panel) and NaPi-IIb in kidney medulla of WT and dKO mice (right panel) show a significant downregulation in NaPi-IIa expression and an unexpected induction of NaPi-IIb transcripts in kidneys of pendrin/NCC-dKO mice. Depiction of immunofluorescence microscopic images demonstrating a significant reduction in the expression of NaPi-IIa and NaPi-IIc in the proximal tubule **(B & C)** of pendrin/NCC-dKO mice (vs. WT animals), and the induction of NaPi-IIb (arrows) in the kidney cortex **(D**, right top panel**)** and medulla **(D**, right bottom panel**)** of pendrin/NCC-dKO animals. Co-localization (middle panel) of NaPi-IIb **(red**, left panel**)** with AQP2 **(green**, right panel**)** in the medullary collecting duct (MCD)-cells of pendrin/NCC-dKO mice is shown **(E)**. (immunofluorescence microscopy results are representative of the staining observed in the kidney sections from at least n = 3 animals/genotype).

Next, we compared the expression of NaPi-IIa and NaPi-IIc in kidneys of WT and pendrin/NCC-dKO mice. We also examined the expression of NaPi-IIb, the intestine specific sodium phosphate transporter in the kidney. Our results indicate a significant down-regulation of NaPi-IIa mRNA (Fig 3A, left panel) and the induction of NaPi-IIb transcript (Fig 3A, right panel) in kidneys of pendrin/NCC-dKO mice. Both NaPi-IIa and NaPi-IIc play important roles in phosphate reabsorption in the kidney proximal tubule [27, 28]. Our immunofluorescence labeling demonstrated a significant reduction in the expression of NaPi-IIa and c, the main phosphate-absorbing transporter in the proximal tubule (Fig 3B & 3C). Immunofluorescence microscopic studies also confirmed the induction of the intestine-specific NaPi-IIb on the apical membrane of medullary collecting duct (MCD) cells of pendrin/NCC-dKO but not WT mice (Fig 3D). Immunofluorescence double staining studies confirmed the co-localization (Fig 3E, **middle panel**) of NaPi-IIb (Fig 3E, **left panel**) with aquaporin-2 (AQP2, Fig 3E, **right panel**) in MCD cells of pendrin/NCC-dKO.

The specificity of anti-NaPi-IIb antibody that were utilized for the above studies was verified by comparing the staining of the small intestines of WT and NaPi-IIb KO mice by immunofluorescence microscopy. Our results (S1 Fig), indicate that NaPi-IIb antibodies strongly stain the apical membrane of the epithelial cells of the small intestine in WT, which is completely absent in NaPi-IIb KO mice.

We have examined the expression of the Na^+^/K^+^ ATPase in the kidneys of WT and pendrin/NCC-dKO mice by immunofluorescence microscopy. The results are included in S2 Fig and show that the intensity of the labeling of Na^+^/K^+^ ATPase is not reduced in kidneys of pendrin/NCC-dKO mice.

**Fig 4:**
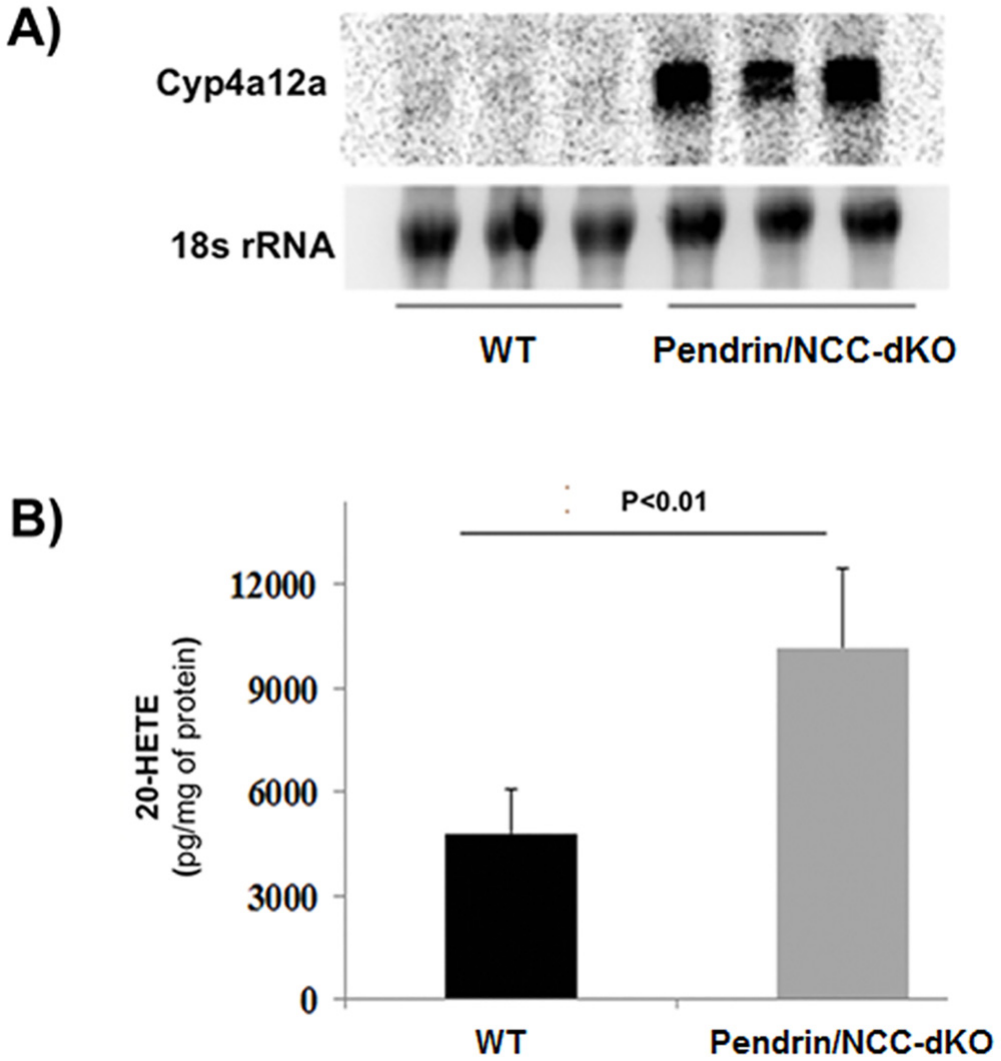
Comparison of renal Cyp4a12a and 20-HETE levels in WT and pendrin/NCC-dKO mice. **(A)** The expression of Cyp4a12a in the kidneys of pendrin/NCC-dKO and WT mice (n = 3) were examined by northern blot analysis. **(B)** Renal 20-HETE levels in WT and pendrin/NCC-dKO mice were compared (n = 3 mice/genotype).

Generation of arachidonic acid pathway metabolites (e.g. 20-HETE and PGE-2) increases in conditions associated with salt wasting and dehydration [29, 30]. We therefore compared the expression of the enzymes involved in the generation of these metabolites in WT versus pendrin/NCC-dKO mice. Our results indicate that the mRNA expression levels of 20-HETE generating enzyme, Cyp4a12a, is significantly increased in kidneys of pendrin/NCC-dKO mice (Fig 4A), which is accompanied by enhanced levels of 20-HETE in the kidney extracts of pendrin/NCC-dKO mice (Fig 4B).

**Fig 5:**
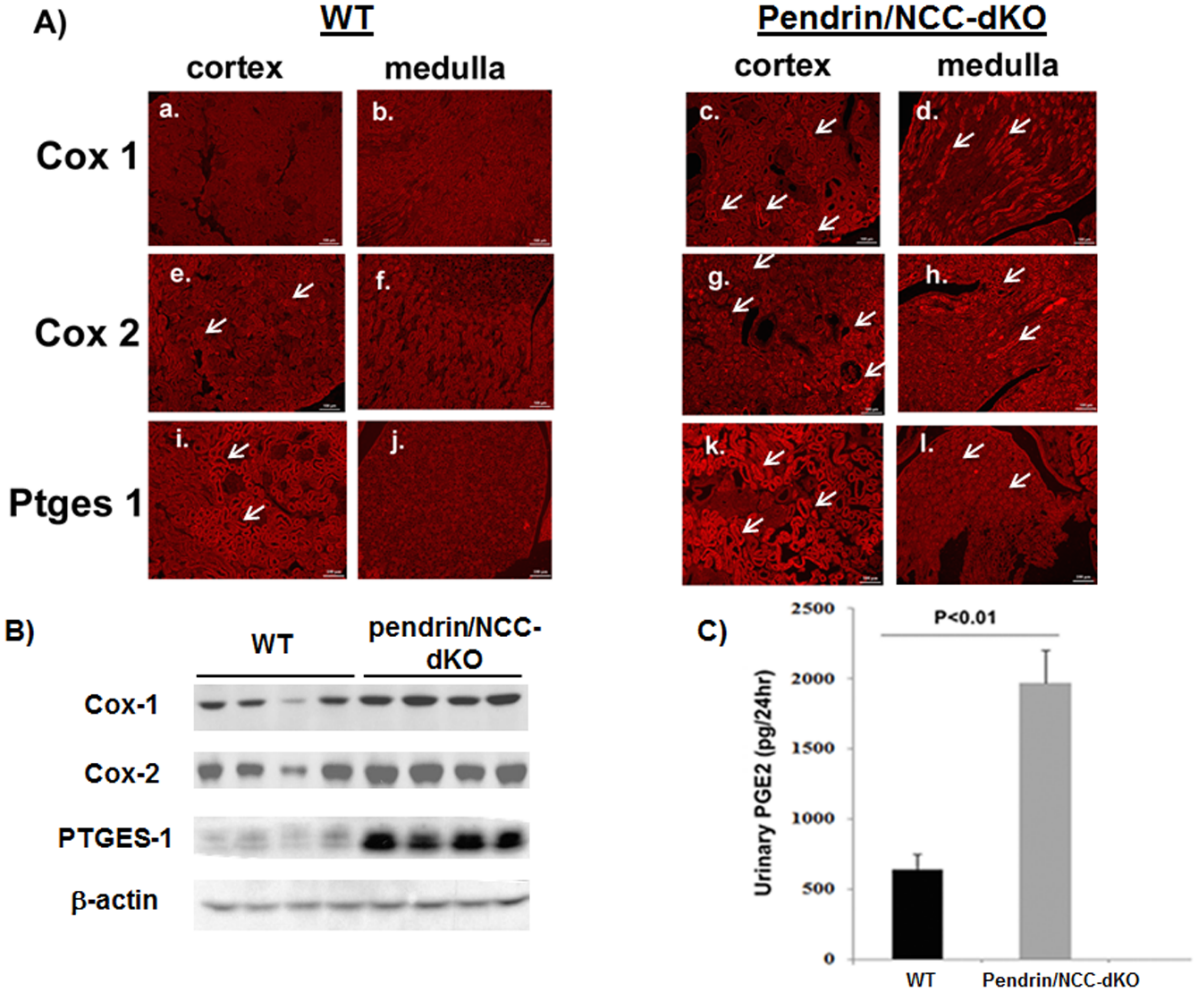
The expression of arachidonic acid pathway enzymes and production of PGE2 are elevated in pendrin/NCC-dKO mice. **(A)** The expression of Cox1 (panels **a-d**), Cox2 (panels **e-h**) and Ptges1 (panels **i-l**) were compared in the kidneys of WT and pendrin/NCC-dKO mice. Arrows point to positive staining tubules **(B)** The expression of Cox1, Cox2 and Ptges1 were examined in the kidneys of pendrin/NCC-dKO and WT mice (n = 4) by western blot analysis (immunofluorescence microscopy results are representative of the staining observed in the kidney sections from n = 3 animals/genotype). **(C)** Urinary PGE-2 levels in WT and pendrin/NCC-dKO mice were compared (results are the average+/-SEM of time matched urine samples from n = 6 animals/genotype).

Immunofluorescence microscopic analysis of WT and pendrin/NCC-dKO mice (Fig 5A) demonstrated that the expression of Cox1 (panels **a-d**), Cox2 (panels **e-h**) and Ptges1 (panels **i-l**) were higher in both the renal cortex and medulla of pendrin/NCC-dKO compared to WT mice. Western blot analyses of kidney extracts further confirmed the above observations (Fig 5B). Examination of urinary PGE-2 levels in WT and pendrin/NCC-dKO mice also revealed that pendrin/NCC dKO had significantly elevated levels of PGE-2 compared to WT mice (Fig 5C).

### Provision of high salt diet corrects the phenotypes of pendrin/NCC-dKO mice

**Fig 6:**
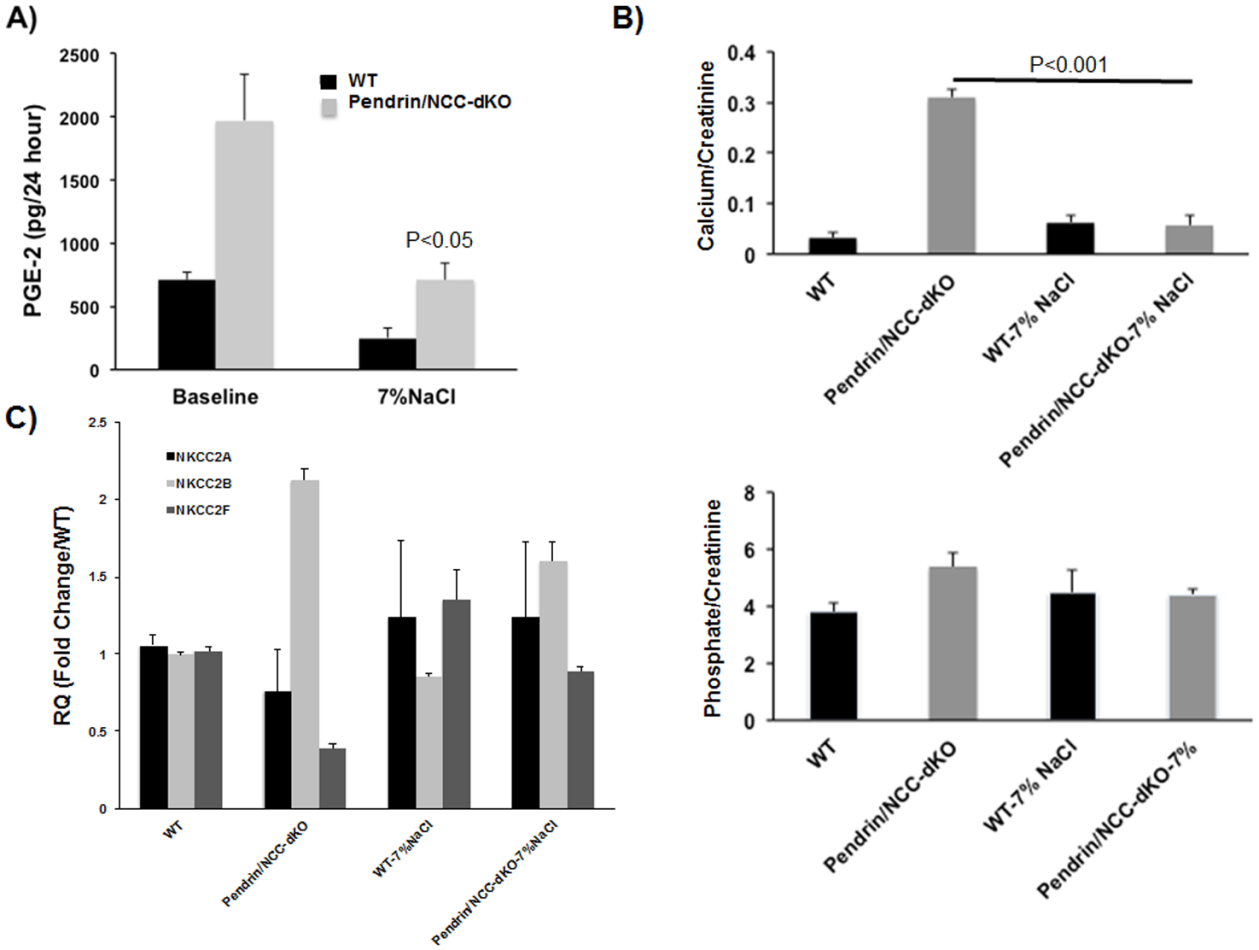
The effect of high salt diet on calcium and phosphate excretion, and the expression of NKCC2 isotype in WT and pendrin/NCC-dKO mice. Effect of provision of liquid normal and high salt diets for 7 days **(A)** on urinary PGE-2 levels, **(B)** calcium and phosphate excretion and **(C)** the expression levels of NKCC2 isotypes in WT and pendrin/NCC-dKO mice was examined in WT and pendrin/NCC-dKO mice (results are the average+/- SEM of n = 6 animals/genotype/treatment for parts A and B; and the average +/- SEM of n = 4 animals/genotype/treatment for part C).

Salt loading alleviates the volume contraction and dampens the activation of RAAS in pendrin/NCC-dKO mice [9]. The correction of volume depletion by increased dietary salt intake (7% salt in diet) for 7 days decreased the urinary PGE-2 levels (Fig 6A) in pendrin/NCC dKO mice (from 1970 +/-467 to 710 +/- 134 pg/24 hr after salt loading, p<0.05). The animals placed on high salt diets also showed reduced calcium and phosphate excretion (Fig 6B) and correction in the expression pattern of NKCC2 isotypes (Fig 6C).

### Indomethacin treatment improves the phenotypic aberrancies in the pendrin/NCC dKO animals

**Fig 7:**
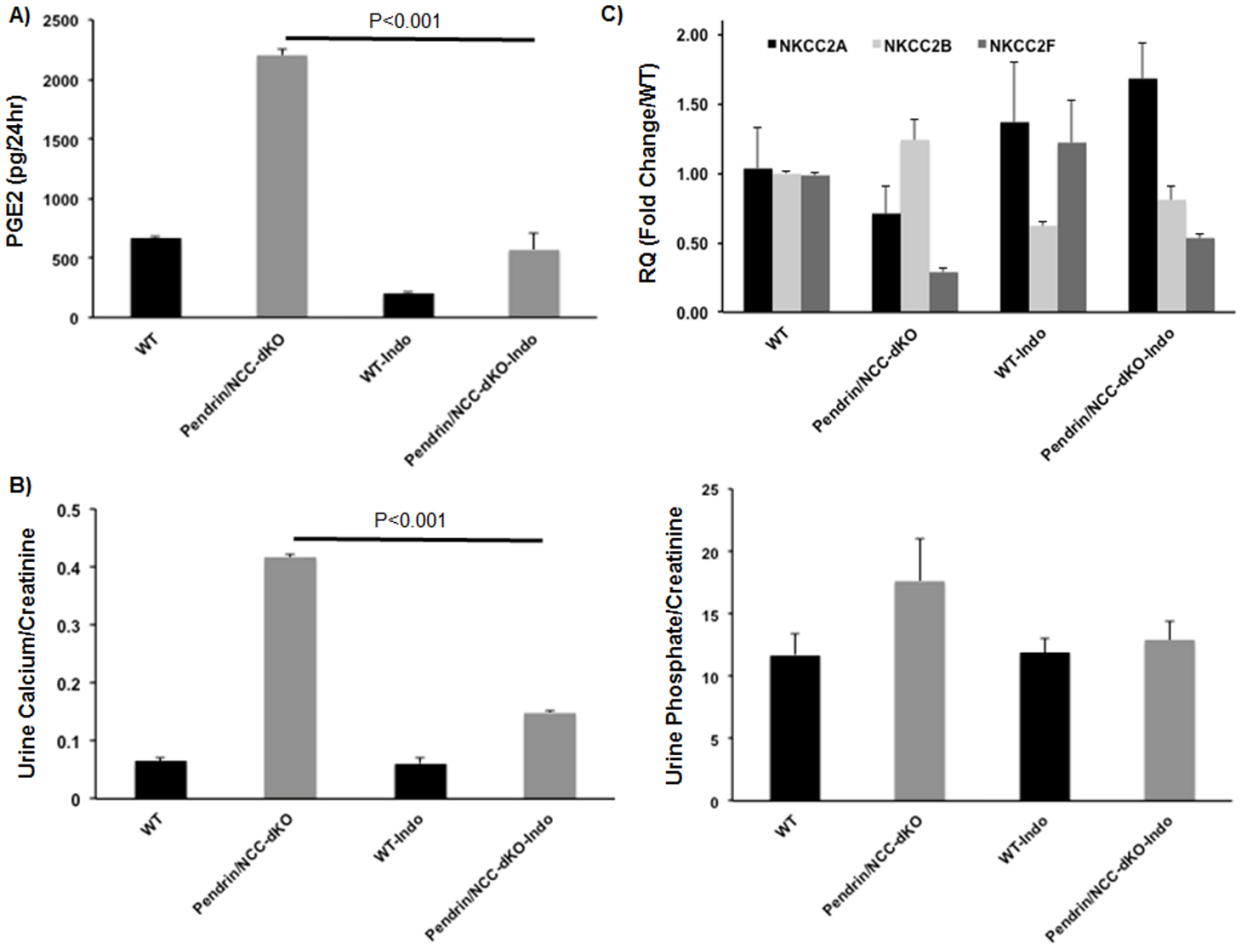
The effect of indomethacin treatment on urine calcium and phosphate excretion, and NKCC2 isotype expression of WT and pendrin/NCC-dKO mice. Effect of indomethacin treatment (25mg/kg BW/day) on **(A)** urinary PGE2 levels, **(B)** calcium and phosphate excretion and **(C)** the expression levels of NKCC2 isotypes was determined (results are the average+/-standard deviation of n = 6 animals/genotype/treatment for parts A and B; and the average +/- SEM of n = 4 animals/genotype/treatment for part C).

Next, we tested the hypothesis that increased generation of arachidonic acid metabolites contributes to the worsening of salt wasting, and enhanced excretion of water and minerals in pendrin/NCC-dKO mice. Treatment of pendrin/NCC dKO mice with the Cox inhibitor, indomethacin (25 mg/kg body weight/day), for 3 days significantly reduced PGE-2 levels in the urine of both WT and dKO mice, confirming the efficacy of the treatment regimen (Fig 7A). The 24hr sodium and calcium excretion was also significantly improved in pendrin/NCC-dKO mice in response to indomethacin treatment (Fig 7B). In addition, indomethacin treatment significantly reversed the derangements in NKCC2 isotypes expression in pendrin/NCC-dKO animals, as shown by a reduction in NKCC2B and an increase in NKCC2A and F levels (Fig 7C). Changes in NKCC2A variant were not significantly different when WT and pendrin/NCC animals were compared.

### Inhibition of 20-HETE generation or the ablation of purinergic signaling by purinergic receptor 2 Y2 (Pr2Y2) did not correct the phenotypic abberancies of pendrin/NCC-dKO mice

Treatment of pendrin/NCC-dKO mice with Cyp4a12 inhibitor, HET0016, did not have a significant effect on urine output and osmolality of pendrin/NCC-dKO mice.

Enhanced signaling via Pr2Y2 plays an important role in the synthesis of PGE-2 [31]. Therefore, we examined the effect of inhibition of Pr2Y2 by suramine in pendrin/NCC-dKO mice or its ablation by generating pr2Y2/pendrin/NCC triple knockout mice on the phenotypic abnormalities caused by simultaneous deactivation of pendrin and NCC. Our observations (data not shown) indicate that neither inhibition nor ablation of Pr2Y2 or inhibition of Pr2Y12 had any significant effect on salt wasting caused by the simultaneous deficiency of pendrin and NCC.

## Discussion

The thiazide sensitive sodium chloride co-transporter, NCC, and the Cl^-^/HCO_3_^-^ exchanger, pendrin, play important roles in the reabsorption of sodium and chloride in the distal nephron under normal conditions and are indispensable for the salvation of these electrolytes under salt restricted conditions [11, 32]. Our studies indicate that simultaneous deletion of pendrin and NCC genes gives rise to animals with phenotypic anomalies (e.g. severe salt wasting, volume depletion and renal failure) that are disproportionate to the physiological function of these molecules [9]. Studies in this manuscript elucidate the renal derangements that give rise to the exaggerated phenotype of pendrin/NCC-dKO mice. They specifically demonstrate how salt wasting originating in the DCT and CCD led to the impairment of electrolyte reabsorption in the entire nephron, including the PT and TALH.

Examination of kidney sections of pendrin/NCC-dKO mice exposed a number of changes including the expansion of JGA and the presence of numerous calcium phosphate deposits in the lumen of the collecting ducts of the pendrin/NCC-dKO mice (Fig 1C). Comparison of the kidneys of WT and pendrin/NCC-dKO mice also revealed that: 1) the isoform composition of NKCC2 (Fig 2) are altered in favor of high Cl^-^ affinity B variant of NKCC2 [26]; 2) the expression of NaPi-IIa and c in the proximal tubule is significantly reduced (Fig 3); and 3) expression of enzymes involved in 20-HETE (Cyp4a12) and PGE-2 (Cox1, Cox2 and Ptges1) synthesis are up regulated in the kidneys of the pendrin/NCC-dKO mice (Figs 4 & 5). These changes led to reduced reabsorption of sodium and chloride, exacerbate salt wasting, and diminish the ability of the kidneys to salvage phosphate and calcium.

Interestingly, serum PTH and FGF-23 levels in WT and pendrin/NCC-dKO mice were similar despite the latter having significantly lower serum calcium and phosphate levels. A similar dissociation between increased calcium excretion and PTH levels has been reported in idiopathic hypercalciuria patients with high propensity to form kidney stones [33]. In addition, studies by Leonhrdt et al and Shoemaker et al indicate that calcium loss in some neonatal Bartter Syndrome patients is independent of serum PTH levels and is most likely mediated through prostanoid generation [6, 34]. The mechanistic basis of how calcium and phosphate homeostasis are decoupled from PTH and FGF23 signaling in pendrin/NCC-dKO mice remains to be elucidated. However, in neonatal Bartter Syndrome patients this decoupling is most likely mediated via a calcitropic substance in their serum and urine, which is not found in normal individuals [34].

The decreased renal calcium reabsorption and hypercalciuria in pendrin/NCC-dKO mice is likely due to the disruption of the charge gradient needed for paracellular movement of calcium in the proximal tubule and the thick ascending limb of Henle. The diminished reabsorption of salt in the TALH is due to a reduction in the expression of NKCC2 isoform F [17, 35]. The pathogenesis of impaired calcium absorption in the proximal tubule in pendrin/NCC dKO mice remains speculative.

The reduction in phosphate reabsorption is due to the downregulation of phosphate transporters NaPi-IIa and c in the proximal tubule of the pendrin/NCC-dKO mice, which is likely caused by enhanced generation of arachidonic acid pathway products [36]. A novel observation in this manuscript is the induction of NaPi-IIb expression in the medullary collecting ducts of pendrin/NCC-dKO mice (Fig 3). Although the induction of NaPi-IIb isoform in the kidney has not been previously reported, our results suggest that these changes may be triggered by the down regulation of phosphate reabsorption in the proximal tubule. In support of this, our results demonstrate that in ischemia/reperfusion injury, the expression of NaPi-IIa is decreased whereas that of NaPi-IIb is induced. The induction of NaPi-IIb in kidneys of pendrin/NCC dKO mice or in mice subjected to ischemic reperfusion injury may be an attempt by the kidney to mitigate the magnitude of phosphate wasting caused by reductions in phosphate reabsorption in the proximal tubule. Our results also indicate that the impaired phosphate reabsorption in the intestine of pendrin/NCC-dKO mice did not play a significant role in reduced serum phosphate levels in these animals since compared to WT mice the expression of NaPi-IIb mRNA in the small intestine of pendrin/NCC dKO mice is only incrementally decreased (S3 Fig). These results demonstrate the presence of a mechanism, where overt damage or dysfunction in the phosphate absorbing machinery of the proximal tubule can lead to adaptive changes in the kidney in order to partially compensate for the loss of expression and/or activity of the normal renal NaPi-ll molecules.

Hyperprostaglandin E syndrome (HPS), which is used synonymously with neonatal Bartter’s Syndrome, is characterized by elevated endogenous PGE-2 synthesis, which is thought to be largely responsible for the aggravation of the clinical symptoms associated with this disorder [6, 7]. Hypercalcuria and nephrocalcinosis are the most evident features of HPS. Studies by Vaisbich et al [37] indicated that a large percentage of patients with neonatal Bartter Syndrome have low serum phosphate levels. Recent studies also show the down regulation of kidney NaPi-IIa in a mouse model of type II Bartter syndrome [38]. These alterations closely resemble our observations in pendrin/NCC-dKO mice and corroborate our findings in the current study. Our present studies challenge the exclusive association of HPS and Bartter’s Syndrome by demonstrating phenotypic similarities between Bartter’s Syndrome [3, 4] and the pendrin/NCC-dKO mouse model [9]. Our results strongly suggest that it is the renal salt wasting followed by volume depletion that is responsible for HPS, and this is independent of the affected nephron segments. Enhanced production of PGE-2 is also detected in a model of salt wasting and volume depletion caused by acetazolamide and hydrochlorothiazide [14], confirming that it is the volume depletion subsequent to salt wasting per se, and not defects in a specific nephron segment, that triggers the generation of PGE-2 and leads to the onset of hypercalciuria. Our results using chemical inhibitors of purinergic receptor signaling or ablating the Pr2Y2 receptor are a strong indication that the flow activation of purinergic signaling is not involved in the mediation of renal alterations in pendrin/NCC-dKO mice.

In support of our proposal, a number of studies indicate that the expression and activity of Cox2 are enhanced in response to engagement of prorenin receptor and directly or indirectly by angiotensin II [39, 40], both of the above paths may play a role in the induction of PGE-2 synthesis in the pendrin/NCC-dKO model. Increased angiotensin II levels can also lead to elevated production of tumor necrosis factor-alpha (TNF-α) [41, 42]. The role of TNF-α in the induction of PGE2 synthesis via up-regulation of Cox2 and Ptges1 is well documented [41, 43, 44]. Interestingly, TNF-α expression is enhanced in kidneys of pendrin/NCC dKO mice (S4 Fig), suggesting that the induction of TNFα, consequent to activation of the RAAS, may be important in increasing the expression of Cox2 and Ptges1 and generation of PGE-2 and mediation of phenotypic anomalies in pendrin/NCC-dKO mice. It is also possible that increased PGE2 levels through EP3 receptor signaling can lead to the activation of inhibitory G protein (Gi) [45]. The active Gi can in turn interfere with the activation of adenylate cyclase AC6, which is implicated in the mediation of V_2_ receptor (V_2_R) signaling [46, 47], by stimulatory G protein. The latter may inhibit the signaling through antidiuretic hormone receptor (V_2_R) and prevent the salvation of water through AQP2. This may also account for the urine-concentrating defect of the pendrin/NCC-dKO mouse [9]. The above results indicate the presence of a non-purinergic-PGE2 dependent pathway in the mediation of renal tubular alterations in pendrin/NCC-dKO mice.

Recent studies indicate that simultaneous deficiency of NaPi-IIa and c in mouse leads to the development of phenotype that resembles hereditary hypophosphatemic rickets with hypercalciuria (HHRH) [48], while in humans mutations in NaPi-IIc are sufficient to cause HHRH [49]. Phenotypic alterations (e.g. reduced expression levels of NaPi-IIa and c) observed in pendrin/NCC-dKO mice further suggest that severe salt wasting in the distal nephron also lead to changes that resemble what is observed in HHRH.

## Conclusion

The results presented here indicate that salt wasting followed by volume depletion activates a cascade of events that lead to the increased generation of the arachidonic acid metabolite, PGE-2 and 20-HETE. Our results further demonstrate that PGE-2 impairs salt and calcium reabsorption in the TALH, as well as phosphate reabsorption in the proximal tubule, leading to phosphate and calcium wasting. Based on our studies the aforementioned events are independent of purinergic signaling and are most likely mediated via RASS induced stimulation of PGE2 synthesis.

## Supporting Information

S1 Fig The specificity of NaPi-IIb antibodies. NaPi-IIb antibodies were used for immunofluorescence microscopy experiments in the small intestine of WT (Right panel) and NaPi-IIb KO mice (left panel). As indicated, NaPi-IIb expression is detected on the apical membrane (villi) of small intestine of WT mice. The labeling by NaPi-IIb antibodies was completely abrogated in small intestines of NaPi-IIB KO mice.
(TIF)

S2 Fig Comparison of the expression of Na^+^/K^+^-ATPase in WT and pendrin/NCC-dKO mice. Immunofluorescence microscopy indicated that the intensity of the Na^+^/K^+^ ATPase labelling is not reduced in kidneys of pendrin/NCC-dKO mice.
(TIF)

S3 Fig The expression of NaPi-IIb transcript in the intestines of WT and pendrin/NCC-dKO mice by northern blot analysis. Our northern blot analyses indicate that compared to WT mice the expression of NaPi-IIb mRNA in the small intestine of pendrin/NCC dKO mice is only incrementally decreased.
(TIF)

S4 Fig Expression of TNF-α is significantly higher in the kidneys of pendrin/NCC-dKO mice. Northern blot analyses indicate that TNF-α expression is enhanced in kidneys of pendrin/NCC dKO mice.
(TIF)
