# Supplementary figures and images for: Prostaglandin-E2 Mediated Increase in Calcium and Phosphate Excretion in a Mouse Model of Distal Nephron Salt Wasting
Source: PLoS One. 2016 Jul 21;11(7):e0159804. doi: 10.1371/journal.pone.0159804 (PMC4956050; doi:10.1371/journal.pone.0159804)

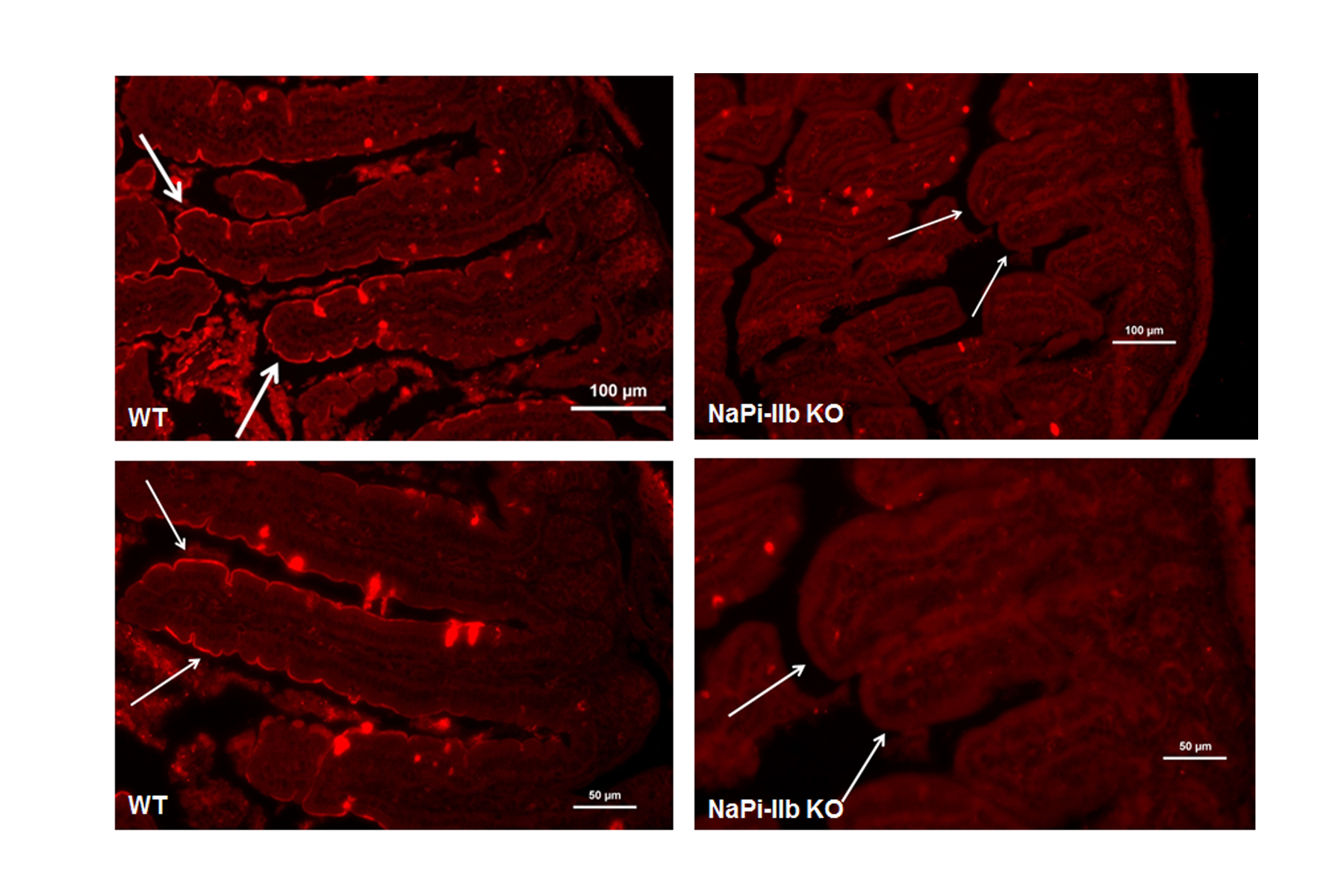

Supplement: S1 Fig — NaPi-IIb antibodies were used for immunofluorescence microscopy experiments in the small intestine of WT (Right panel) and NaPi-IIb KO mice (left panel). As indicated, NaPi-IIb expression is detected on the apical membrane (villi) of small intestine of WT mice. The labeling by NaPi-IIb antibodies was completely abrogated in small intestines of NaPi-IIB KO mice. (TIF) [file pone.0159804.s001.tif]

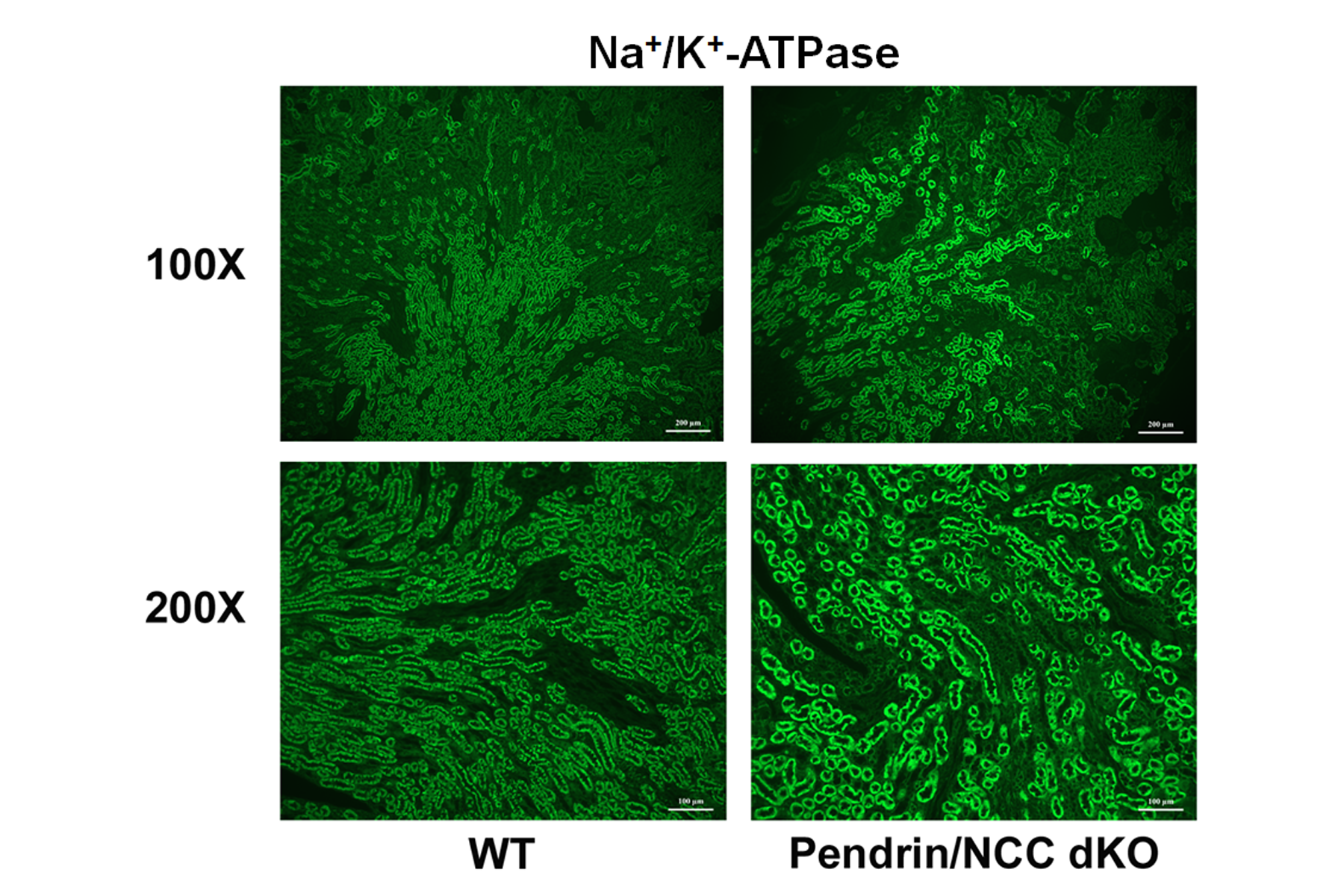

Supplement: S2 Fig — Immunofluorescence microscopy indicated that the intensity of the Na+/K+ ATPase labelling is not reduced in kidneys of pendrin/NCC-dKO mice. (TIF) [file pone.0159804.s002.tif]

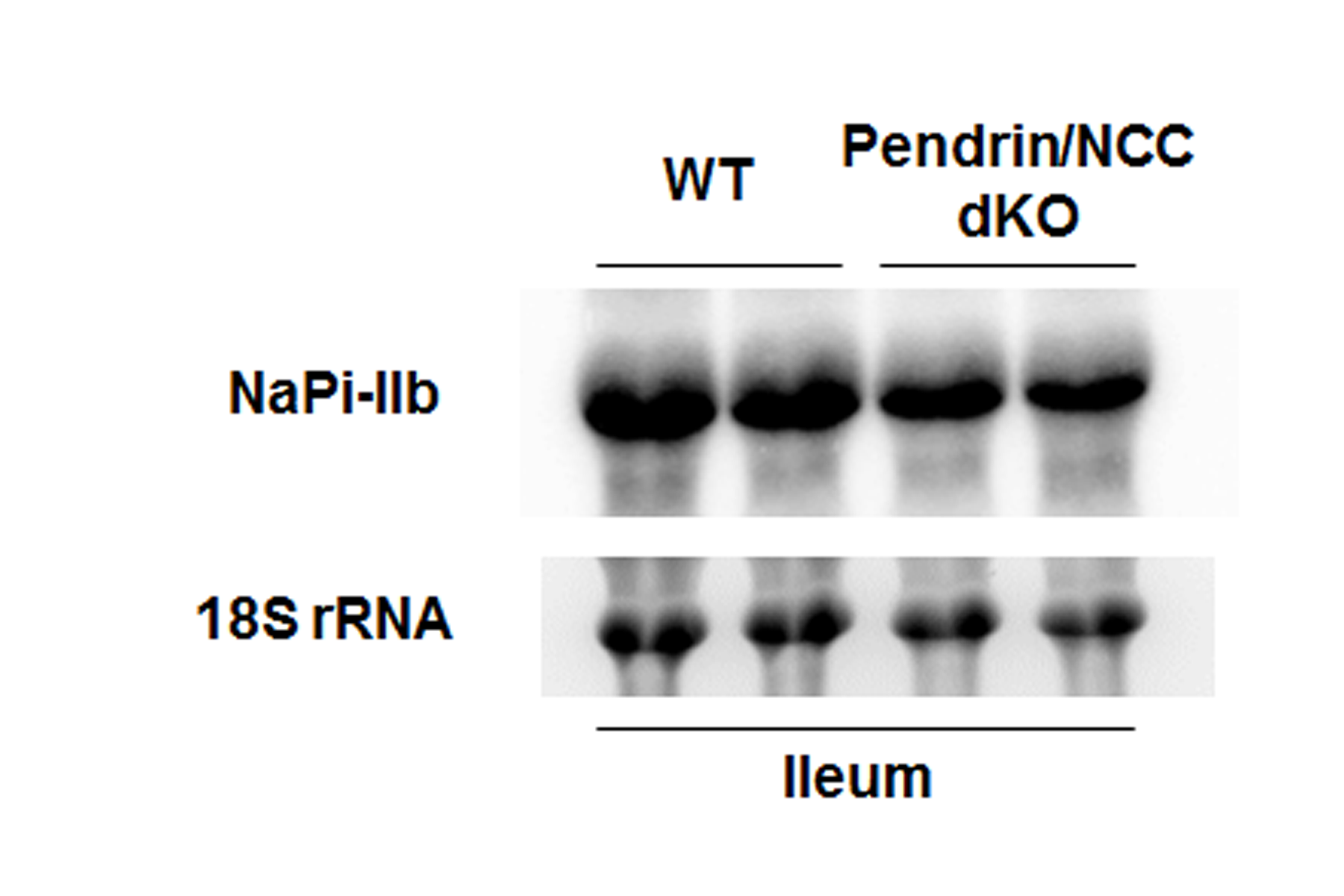

Supplement: S3 Fig — Our northern blot analyses indicate that compared to WT mice the expression of NaPi-IIb mRNA in the small intestine of pendrin/NCC dKO mice is only incrementally decreased. (TIF) [file pone.0159804.s003.tif]

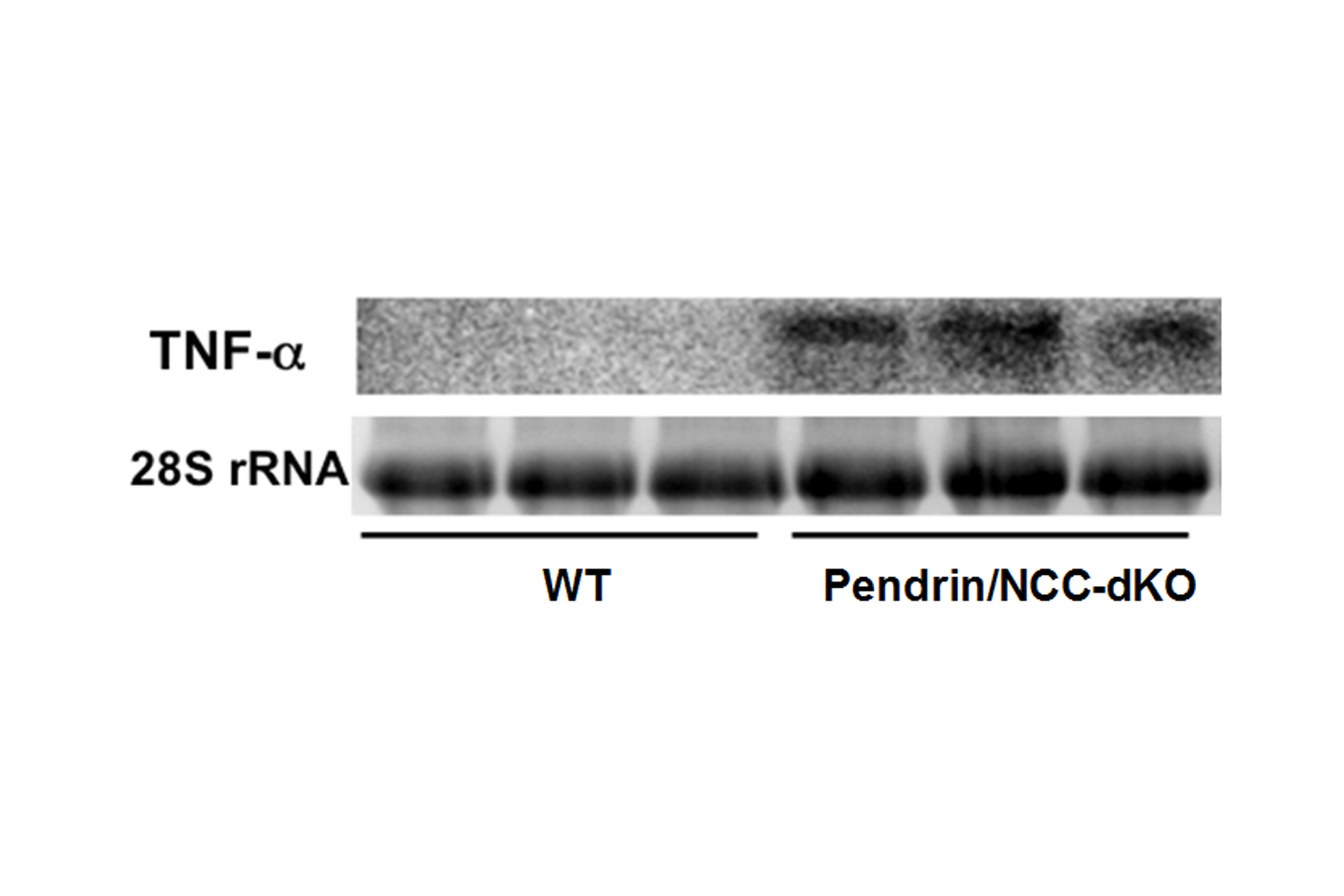

Supplement: S4 Fig — Northern blot analyses indicate that TNF-α expression is enhanced in kidneys of pendrin/NCC dKO mice. (TIF) [file pone.0159804.s004.tif]
